# PubChem3D: Conformer generation

**DOI:** 10.1186/1758-2946-3-4

**Published:** 2011-01-27

**Authors:** Evan E Bolton, Sunghwan Kim, Stephen H Bryant

**Affiliations:** 1 National Center for Biotechnology Information National Library of Medicine National Institutes of Health Department of Health and Human Services 8600 Rockville Pike, Bethesda, MD 20894, USA

## Abstract

**Background:**

PubChem, an open archive for the biological activities of small molecules, provides search and analysis tools to assist users in locating desired information. Many of these tools focus on the notion of chemical structure similarity at some level. PubChem3D enables similarity of chemical structure 3-D conformers to augment the existing similarity of 2-D chemical structure graphs. It is also desirable to relate theoretical 3-D descriptions of chemical structures to experimental biological activity. As such, it is important to be assured that the theoretical conformer models can reproduce experimentally determined bioactive conformations. In the present study, we investigate the effects of three primary conformer generation parameters (the fragment sampling rate, the energy window size, and force field variant) upon the accuracy of theoretical conformer models, and determined optimal settings for PubChem3D conformer model generation and conformer sampling.

**Results:**

Using the software package OMEGA from OpenEye Scientific Software, Inc., theoretical 3-D conformer models were generated for 25,972 small-molecule ligands, whose 3-D structures were experimentally determined. Different values for primary conformer generation parameters were systematically tested to find optimal settings. Employing a greater fragment sampling rate than the default did not improve the accuracy of the theoretical conformer model ensembles. An ever increasing energy window did increase the overall average accuracy, with rapid convergence observed at 10 kcal/mol and 15 kcal/mol for model building and torsion search, respectively; however, subsequent study showed that an energy threshold of 25 kcal/mol for torsion search resulted in slightly improved results for larger and more flexible structures. Exclusion of coulomb terms from the 94s variant of the Merck molecular force field (MMFF94s) in the torsion search stage gave more accurate conformer models at lower energy windows. Overall average accuracy of reproduction of bioactive conformations was remarkably linear with respect to both non-hydrogen atom count ("size") and effective rotor count ("flexibility"). Using these as independent variables, a regression equation was developed to predict the RMSD accuracy of a theoretical ensemble to reproduce bioactive conformations. The equation was modified to give a minimum RMSD conformer sampling value to help ensure that 90% of the sampled theoretical models should contain at least one conformer within the RMSD sampling value to a "bioactive" conformation.

**Conclusion:**

Optimal parameters for conformer generation using OMEGA were explored and determined. An equation was developed that provides an RMSD sampling value to use that is based on the relative accuracy to reproduce bioactive conformations. The optimal conformer generation parameters and RMSD sampling values determined are used by the PubChem3D project to generate theoretical conformer models.

## Background

PubChem [1-4] is an open archive for the biological activities of small molecules. It consists of three primary databases: Substance, Compound, and BioAssay. The PubChem Compound database contains the unique chemical structure content found in the PubChem Substance database. When possible, a theoretical 3-D conformer model description is generated for each and every record in the PubChem Compound database. This 3-D layer is the basis of the PubChem3D project.

PubChem provides search and analysis tools to assist users in locating desired information in the archive. The importance of this cannot be understated with more than 70 million substance descriptions, 28 million unique small molecules, 480,000 biological assays, and 110 million biological assay outcomes (results from a substance tested in an assay is considered an outcome). Nearly all of these tools focus on the notion of chemical structure similarity at some level. PubChem3D enables similarity of chemical structure 3-D conformers to augment the existing similarity of 2-D chemical structure graphs.

With the goal in mind to use theoretical 3-D descriptions of chemical structures to relate experimental biological activity, there must be an appropriate determination whether these constructs have any relevance to reality. Presumably, if the 3-D conformer model can readily reproduce a reputed "experimental bioactive ligand" conformation with sufficient regularity, one tends to feel (more) confident that the theoretical methodology may be producing biologically meaningful results. There is currently no way to prove with any absolute degree of certainty that all theoretical conformers produced will be biologically relevant; however, one can check if all known experimental "bioactive" conformers of a chemical structure can be found in its theoretical model.

The largest publicly available source of "experimental" 3-D coordinates of chemical structures is the Protein Data Bank (PDB) [5]. This data is not without its considerable issues [6-10]. Most "experimental" 3-D coordinates for small molecules provided by the PDB are, in essence, theoretical models derived from fitting electron density produced by X-ray diffraction experiments to the presumed location of atoms that are part of a protein, a ligand (typically bound to the protein), and other moieties (ions, water molecules, etc.). At times, electron density is lacking or there is some degree of uncertainty as to the precise location of the small molecule atoms. In this context, the ligand location or protein binding geometry cannot be considered to be well understood, with many possible conformations of the same ligand plausible [6,11]. These concerns will be largely ignored here and all the PDB ligands will be treated as experimental fact for the purposes of this study.

There are a number of established theoretical conformer generator packages available [11-16]. Many of these perform reasonably well [6,17,18], being both fast and accurate. The specific requirements of the PubChem3D project (such as a multiplatform programmatic interface) made the choice of one of these (OMEGA [19]) very easy. Considering the size of PubChem, the need to relate similar conformers, and the desire to allow users to analyze biological activity patterns in real-time, it requires all 3-D information to be pre-computed and stored. This requirement is a primary limiting factor.

Conformer generator packages are very capable to produce many conformers per chemical structure. This is important as a small molecule of reasonable flexibility at room temperature can access many potential conformational shapes; however, it is impractical to store all produced theoretical conformations per chemical structure, especially when you need to consider the storage requirements for millions of compounds. A common practice is to limit the conformer count using some mix of energy-based filtering, minimum conformer root-mean-squared distance (RMSD) of pair-wise atoms, and random sampling. This leaves one to determine how best to minimize the count of conformations stored while not sacrificing coverage or resolution of biologically meaningful conformer space.

In this work, one of a series covering the PubChem3D project, we attempt to answer questions regarding conformer model construction relative to the ability to reproduce PDB ligand 3-D coordinates. For example, what is the base-line conformer generation software accuracy as a function of molecular size and flexibility? Given that conformer models are produced in vacuum, is it beneficial to remove bias towards conformers with intra-molecular interaction to improve accuracy? Is energy filtering useful? What are some practical limitations when generating conformers of flexible molecules? Can one predict average theoretical conformer model accuracy? How do you minimize the count of conformers without significantly impacting accuracy? Using PDB ligand 3-D coordinates, key parameters of conformer model creation are explored to answer these questions. In the process of doing so, a useful relationship is developed relating the size and flexibility of a molecule to the accuracy of reproduction. Further examination is given to accuracy as it relates to limiting the total count of conformers considered in such a model.

## Results and Discussion

### 1. Molecular size and flexibility of the PDB ligands

**Figure 1:**
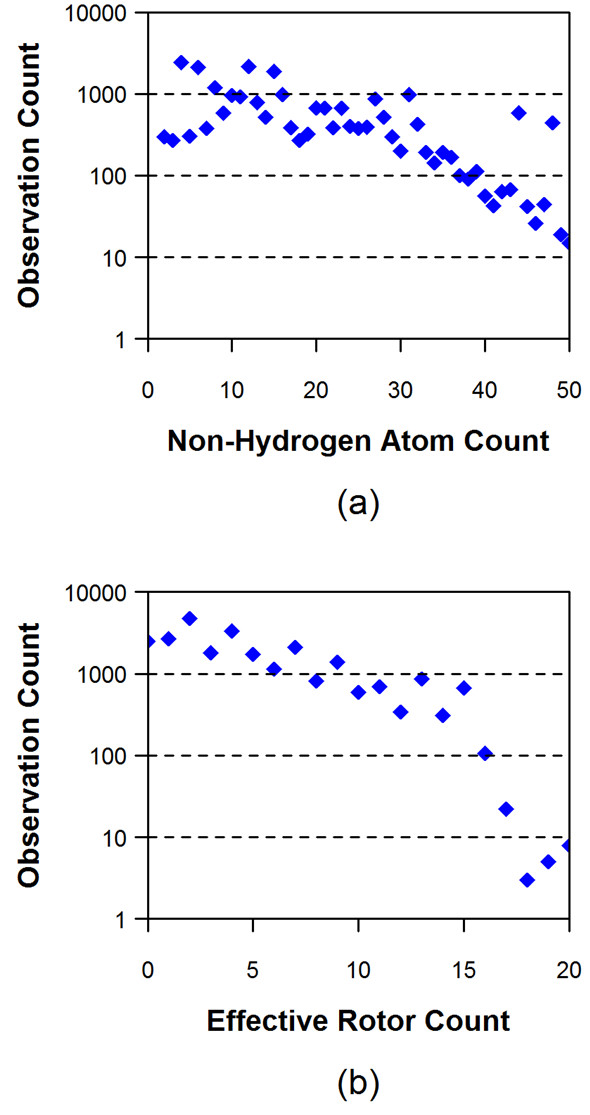
**Distribution of (a) the non-hydrogen atom count and (b) the effective rotor count, binned by whole numbers, for the 25,972 experimentally determined structures in the MMDB ligand data set**.

The size and flexibility of a molecule are important factors affecting the conformer model for a molecule. While the molecular size is approximated by the number of non-hydrogen atoms in the molecule, the molecular flexibility can be expressed in terms of the number of effective rotors [20], which is given as the following:

(1) $$N_{ER}=N_{R}+\frac{N_{NARA}}{5}$$

where *N*_*ER *_is the number of effective rotors, *N*_*R *_is the number of rotatable bonds, and *N*_*NARA *_is the number of "non-aromatic" *sp*^3^-hybridized ring atoms. Note that the value of *N*_*ER *_is not necessarily an integer, but, in this study, is frequently rounded to the nearest whole number. Effective rotors take into account the flexibility of rings as well as rotatable bonds. Figure 1 shows the distributions of the non-hydrogen atom counts and the effective rotor counts (binned by whole numbers) for the experimental structures in the Molecular Modeling Database (MMDB) [21,22] ligand dataset as downloaded from the PubChem Substance database (the MMDB contains only experimentally determined data found in the PDB). On average, the molecules in the MMDB ligand set have 17.1 non-hydrogen atoms and 4.9 effective rotors. Although molecules with up to 50 non-hydrogen atoms are considered in the present study, ~90% of them have 30 or less non-hydrogen atoms. In addition, as shown in panel (b) of Figure 1, molecules with greater than 16 effective rotors rarely occur, due to limiting the MMDB dataset to 15 rotatable bonds.

### 2. Parameter validation for conformer generation

OMEGA [19] was used in the present study. It is known to be among the fastest and most accurate conformer generation programs [17] available. In addition to high quality, it was the only commercially available program that had a non-windows-only C++ application programming interface (API) at the time of project initiation, a critical consideration given the computing environments at the National Center for Biotechnology Information (NCBI). A brief overview of the conformer generation algorithm of OMEGA is given in the *Materials and Methods *section. A more detailed explanation can be found elsewhere [11,23].

OMEGA has many adjustable parameters to generate 3-D conformations with particular attributes. Some important ones are listed in Table 1. To find an optimal set of values, the effects of primary parameters upon conformer generation were tested: (1) the fragment sampling rate for determination of fragment conformation, (2) the type of molecular force field used for the model building and torsion search, and (3) the size of the energy window that determines the energy range of conformers generated. As detailed in the *Materials and Methods *section, only a maximum of 100,000 conformations were considered for a given molecule, meaning that the conformer space of some chemical structures was not fully explored due to this "100-k limit." Therefore, the occurrence of such cases was considered while testing optimal values of parameters.

**Table 1:** OMEGA parameters modified or non-default during conformer generation

Flag	Options	Description
-startfact	20, 50	Determines the fragment sampling rate for determination of fragment conformation [default = 20].

-buildff^*a*^	MMFF94s MMFF94s_NoEstat MMFF94s_Trunc	Specifies the type of a molecular force field used in the model building step [default = MMFF94s_NoEstat]

-searchff^*a*^	MMFF94s MMFF94s_NoEstat MMFF94s_Trunc	Specifies the type of a molecular force field used in the torsion search step [default = MMFF94s_NoEstat].

-ewindow	integers between 1 and 30	Determines the size of the energy window [default = 10.0]. (1) In the model building step: determines the maximum energy range of a ring system. (2) In the torsion search step: determines the maximum energy range of a conformer relative to that of a global minimum conformer.

-MaxConfsGen	100,000	Controls the maximum number of fully constructed conformers that OMEGA will attempt to build [default = 30,000].

-MaxConfs	100,000	Controls the maximum number of conformations to be retained [default = 20,000].

-MaxRotors	15	Controls the maximum number of rotatable bonds in a molecule. If a molecule has more rotatable bonds than this cutoff, OMEGA will not search for conformers [default = 12].

-MaxSearchTime	180	Limits the maximum amount of time (in seconds) spent generating conformers for each molecule [default = 30].

-DefaultRMSD	0.0	Controls the threshold of the RMS distance to determine duplicate conformations. The value of 0.0 indicates that the duplicate detection is skipped [default = 0.8].

^*a *^MMFF94s = the 94s variant of Merck molecular force field; MMFF94s_NoEstat = MMFF94s without coulombic terms; MMFF94s_Trunc = MMFF94s without coulombic and van der Waals attractive terms.

In addition to the non-hydrogen atom pair-wise root-mean-square distance (henceforth termed simply RMSD), the Shape-Tanimoto (ST) value was also used as measure of the accuracy of the conformer models. The ST value between any two molecules A and B is given by the following equation:

(2) $$ST=\frac{V_{AB}}{V_{AA}+V_{BB}-V_{AB}}$$

where *V*_*AA *_and *V*_*BB *_are the self-overlap volume of A and B, respectively, and *V*_*AB *_is the overlap volume between A and B [24-26]. Note that the ST score is a molecular similarity measure ranging from 0 (for no similarity) to 1 (for identical molecules), whereas the RMSD value is a molecular dissimilarity measure ranging from 0 (for identical molecules) to infinity. Among all theoretical 3-D conformers generated for a given molecule, the one with the least RMSD and the one with the greatest ST to the experimental 3-D coordinates were considered to be the most similar to the "bioactive" conformation. Therefore, the accuracy of a conformer model generated by OMEGA was evaluated using the minimum RMSD and the maximum ST values between the experimental conformation and a single theoretical 3-D conformation in the conformer model. In further discussion, the RMSD and ST values *for a conformer model *indicates the RMSD and ST values *between the corresponding experimental structure and the most similar theoretical conformer in the conformer model*, respectively.

#### 2.1. Effects of fragment sampling rate

**Figure 2:**
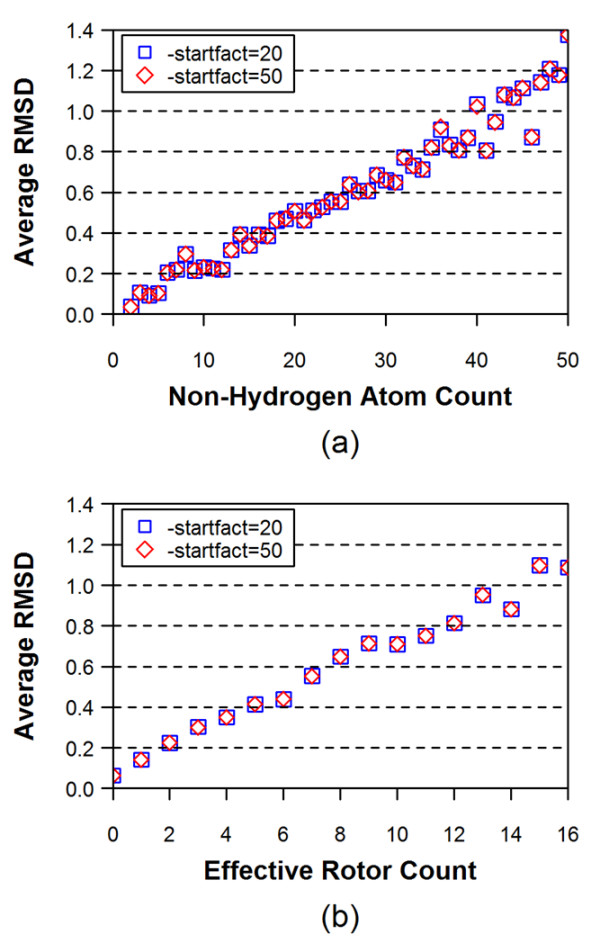
**Effects of the fragment sampling rate upon the average RMSD as a function of (a) non-hydrogen atom count and (b) effective rotor count**. The MMFF94s_NoEstat and MMFF94s_Trunc force fields were used for the model building and torsion search stages, respectively, and the energy window of 15 kcal/mol was used for both stages.

Fragment sampling in the OMEGA model building stage helps to ensure that flexible ring systems are appropriately examined to find all unique ring conformations. In OMEGA, a float-point value for the "-startfact" option determines the fragment sampling rate, using the following equation [23]:

(3) $$N_{samples}=[\text{1}0+[\Sigma \left(nrb=\text{3}\right)-\Sigma \left(nrb=\text{4}\right)]\times \text{startfact}]\;$$

where *N*_*samples *_is the number of samples, and Σ(*nrb *= 3) and Σ(*nrb *= 4) are the sums of the number of atoms in the molecule that have three and four ring bonds, respectively. To investigate effects of the fragment sampling rate upon conformer generation, the conformer models generated using the default value of 20.0 for the "-startfact" option were compared with those generated using the value of 50.0, and the results were summarized in Table 2. In general, using the value of 50.0 rather than the default (= 20.0) was found not to have any significant effect on the overall average RMSD and ST values between the computationally generated conformers and the experimentally determined structures. There were also no significant effects of the increased sampling rate upon the number of conformers generated and the 100-k limit counts. A similar insensitiveness to the fragment sampling rate was observed in Figure 2, which shows the average RMSD as a function of the non-hydrogen atom count and the binned effective rotor count. Thus, the default fragment sampling rate was deemed to be sufficient and the default value of 20.0 was used in the remainder of this study.

**Table 2:** The average root-mean-square distance (RMSD), the average Shape-Tanimoto (ST), the average number of conformers per chemical structure, and the count of the 100-k limit cases for conformer models for all 25,972 3-D reference structures

**Energy Window**^***a***^					5/5					5/15		10/10	10/15	15/15
**Conformer FF**^***b***^		**Full**			**NoEstat**			**Trunc**		**Trunc**		**NoEstat**	**NoEstat**	**NoEstat**

**Fragment FF**^***b***^	**Full**	**NoEstat**	**Trunc**	**Full**	**NoEstat**	**Trunc**	**Full**	**NoEstat**	**Trunc**	**Full**	**Trunc**	**Trunc**	**Trunc**	**Trunc**

**Sampling rate = 20**														
**RMSD**	0.69	0.69	0.71	0.48	0.48	0.48	0.467	0.467	0.462	0.41	0.41	0.42	0.40	0.42
**ST**	0.89	0.89	0.89	0.93	0.93	0.93	0.93	0.93	0.93	0.95	0.95	0.94	0.943	0.94
**# Conformers**	698	698	645	2827	2,814	2,870	5,369	5,352	5,852	12,957	13,012	10,794	13,118	10,794
**# 100-k Limit cases**	23	18	36	94	93	118	216	211	239	2,203	2,209	1,842	2,198	1,842

**Sampling rate = 50**														
**RMSD**	0.69	0.69	0.71	0.48	0.48	0.48	0.466	0.467	0.462	0.41	0.41	0.42	0.40	0.42
**ST**	0.89	0.89	0.89	0.93	0.93	0.93	0.93	0.93	0.93	0.95	0.95	0.94	0.946	0.94
**# Conformers**	696	697	646	2,834	2,818	2,875	5,377	5,358	5,857	12,981	13,025	10,796	13,124	10,796
**# 100-k Limit cases**	21	18	36	96	96	119	217	211	239	2,203	2,210	1,841	2,196	1,841

^*a *^The first and second numbers indicate the energy windows used for model building and torsion search, respectively.

^*b*^Full = the 94s variant of the Merck molecular force field (MMFF94s); NoEstat = MMFF94s without coulombic interaction terms; Trunc = MMFF94s without coulombic and van der Waals attractive terms.

#### 2.2. Effects of force field choice

OMEGA has several pre-defined molecular force fields and it is possible to choose different force fields for model building and torsion search, using the "-buildff" and "-searchff" options, respectively. Three force fields were tested: (1) the 94s variant of the Merck molecular force field (MMFF94s_Full) [27-33], (2) MMFF94s without coulombic interaction terms (MMFF94s_NoEstat), and (3) MMFF94s without coulombic interaction terms and without the attractive part of the van der Waals interaction terms (MMFF94s_Trunc). The latter two force-field variants attempt to remove a perceived bias towards conformers that lower their energy by *intra-molecular *interactions, which are assumed to not be significant when making *inter-molecular *interactions. This consideration is critical as conformer generation is performed in vacuum, which is a very different environment than a protein binding pocket. Varying the force-field terms allows this hypothesis to be tested as improved accuracy should be found if intra-molecular interactions are removed.

Table 3 shows the effects of the force field employed upon the overall average RMSD and ST of the conformer models at increasing energy window values. The type of fragment force field used during the model building stage caused minor variations (less than 0.01) in the average RMSD of conformer models for energy windows 5 and 10 kcal/mol and these disappear entirely at 15 kcal/mol, indicating an overall insensitivity to the type of fragment force field used. Relative to the earlier hypothesis, this suggests that the fragments produced during model building phase were too small to have intra-molecular interactions. On the contrary, at the energy window of 5, 10, and 15 kcal/mol, the overall average RMSD of the conformer model generated using MMFF94s_NoEstat for the torsion search force-field was smaller by 0.21, 0.10, and 0.06, respectively, than those that used MMFF94s_Full, indicating that exclusion of electrostatic terms from the MMFF94s_Full increased the overall average accuracy of the conformer models significantly, but less so as energy window increased. However, almost no perceptible changes in RMSD or ST averages were found upon the additional removal of van der Waals attractive terms, as shown by nearly imperceptible changes in MMFF94s_NoEstat and MMFF94s_Trunc results.

**Table 3:** Overall effects of the choice of the force field and energy window upon the average RMSD and ST values between the computationally generated conformations and the experimental conformations for the 25,972 3-D reference structures

		**Torsion Search Force Field**^***a***^					
		
Energy window (kcal/mol)	**Fragment Force Field**^***a***^	Full		NoEstat		Trunc	
				
		RMSD	ST	RMSD	ST	RMSD	ST
5	Full	0.69	0.89	0.48	0.93	0.47	0.93
	NoEstat	0.69	0.89	0.48	0.93	0.47	0.93
	Trunc	0.71	0.89	0.48	0.93	0.46	0.93

10	Full	0.52	0.92	0.42	0.94	0.42	0.94
	NoEstat	0.52	0.92	0.42	0.94	0.42	0.94
	Trunc	0.52	0.92	0.41	0.94	0.42	0.94

15	Full	0.46	0.93	0.40	0.94	0.40	0.95
	NoEstat	0.46	0.93	0.40	0.94	0.40	0.95
	Trunc	0.46	0.93	0.40	0.94	0.40	0.95

^*a *^Full = the 94s variant of the Merck molecular force field (MMFF94s); NoEstat = MMFF94s without coulombic interaction terms; Trunc = MMFF94s without coulombic and van der Waals interaction terms.

One potential explanation for the higher accuracy of the conformer models generated using the MMFF94s_NoEstat and MMFF94s_Trunc force fields arises from their ability to provide more conformations than MMFF94s in the same energy window threshold. The MMFF94s_Full force field includes additional terms that can lower the energy of conformations with intra-molecular interactions. Removal of such force field terms can increase the energy of conformers with intra-molecular interactions, allowing conformers without these interactions to be represented in a conformer model. This explanation is consistent with the data in Table 4, which shows that MMFF94s_NoEstat and MMFF94s_Trunc produced significantly more conformers per compound on average than MMFF94s_Full. The increased number of conformers per molecule conceptually gives a better chance to have a conformer close to an experimentally determined structure, resulting in the smaller RMSD and greater ST values in Table 3. Because of this, however, the MMFF94s_NoEstat and MMFF94_Trunc were also found to more frequently result in 100-k limit cases. Considering that each 100-k limit case suggests a truncation of energetically possible conformations, their substantial frequency increase (by about a factor of five) as a function of increasing energy window may play a role in the reduction of RMSD differences between MMFF94s_Full force field and MMFF94s_NoEstat variants.

**Table 4:** The overall average conformer count per molecule and 100-k limit count for different force field types and energy window sizes for the 25,972 3-D reference structures

Energy Window (kcal/mol)	**Force Field**^***a***^		
	
	Full	NoEstat	Trunc
	**Conformer count**		
5	698	2,818	5,857
10	2,817	10,852	10,889
15	5,599	13,219	13,178
	**100-k limit count**		
5	21	96	239
10	250	1,788	1,844
15	491	2,189	2,220

^*a *^Full = the 94s variant of the Merck molecular force field (MMFF94s); NoEstat = MMFF94s without coulombic interaction terms; Trunc = MMFF94s without coulombic and van der Waals interaction terms.

Another potential explanation for the superiority of MMFF94s_NoEstat and MMFF94_Trunc over the MMFF94s_Full force field is that the lack of intra-molecular interaction is an important characteristic of a biologically relevant conformation of a small-molecule ligand found in its complex with the protein. When a small-molecule ligand binds to its target protein, the intra-molecular interaction in the ligand molecule that exists in its unbound state is likely to disappear because of a conformational change which enhances inter-molecular interaction between the molecule and the target protein. Regardless of the exact reason why, employing the MMFF94s_NoEstat for conformer model generation appears to be a more sensible choice than the MMFF94s_Full due to the accuracy improvement.

Overall, it appears that removal of electrostatic terms has a rather favorable effect on improving overall accuracy of reproduction of experimental bound ligand geometries. Removal of attractive van der Waals terms does not appear to have any significant effect. As such, the remainder of this study will only consider the MMFF94s_NoEstat force field variant.

#### 2.3. Effects of energy window

**Figure 3:**
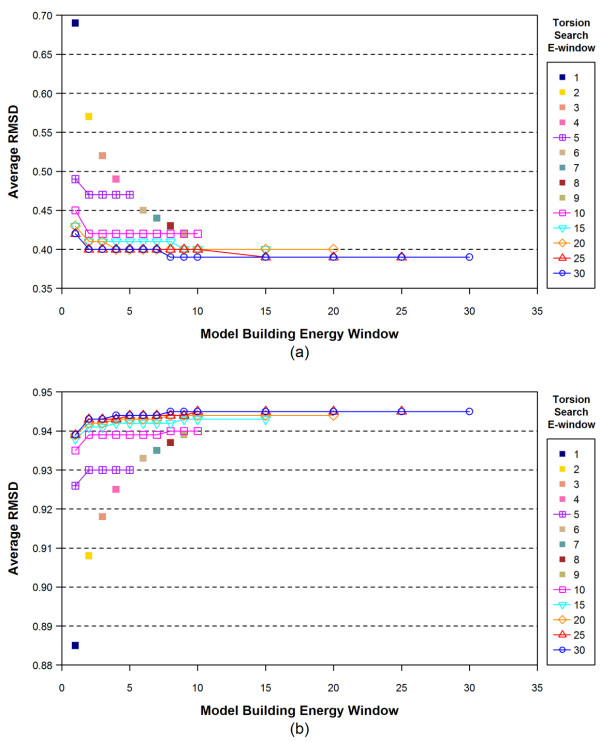
**The overall average RMSD and Shape-Tanimoto (ST) values of the conformer models for all 25,972 structures as a function of the model building energy window and the torsion search energy window (in kcal/mol)**.

In the model building step, the energy window, specified with the "-ewindow" flag, primarily limits the ring conformations allowed in the torsion search step. If the strain energy of a ring conformation is greater than the least-energy ring conformation plus the value of the energy window, then the ring conformation is discarded. When this "-ewindow" parameter is used in the torsion search step, the energy window determines the maximum energy range of conformers, relative to the global-minimum conformer. Figure 3 shows the effect of energy windows on the overall average accuracy of the conformer models generated. When both the model building and torsion search energy windows were 1 kcal/mol, the overall average RMSD and ST of the conformer model generated were 0.69 and 0.885, respectively. On the other hand, the use of an energy window for both stages of 30 kcal/mol resulted in a substantially improved overall average RMSD of 0.39 and an ST of 0.945. This indicates that a larger energy window gives more accurate conformer models, consistent with the data in Table 3. The increased energy window allows more conformational diversity of a molecule to be considered, but also results in more conformers per molecule (and more 100-k limit cases), as shown in Table 4. Note in Figure 3 that a rapid near-convergence in the overall average RMSD and ST occurs at the energy windows of 10 and 15 kcal/mol for model building and torsion search, respectively. The overall average RMSD and ST at these energy windows were 0.40 and 0.944, respectively. Employing bigger energy windows provided only small improvements to the overall accuracy of the conformer models. Therefore, when looking at overall average results, it initially appears reasonable to use an energy window of 10 kcal/mol for model building and 15 kcal/mol for torsion search without significant reduction in overall accuracy.

### 3. Accuracy of conformer models and 100-k limit cases

**Figure 4:**
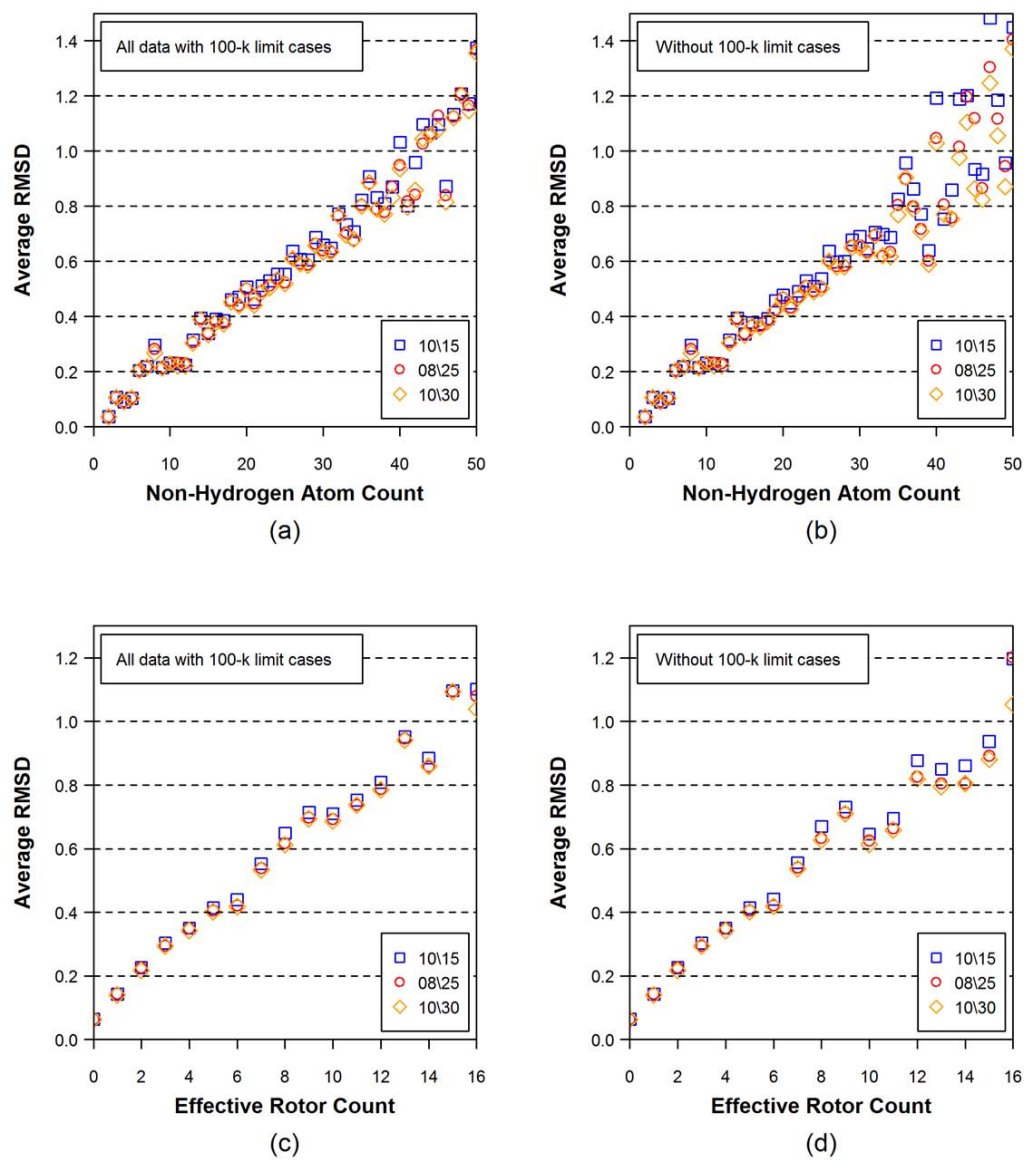
**Average RMSD values for all 25,972 3D reference structures as a function of non-hydrogen atom count [(a) and (b)] and effective rotor count [(c) and (d)]**. In Panels (b) and (d), the 100-k limit cases, in which the number of conformers reached the maximum number allowed, were removed. The numbers in the legend boxes indicates the energy-window values used in the model building (first) and torsion search (second) stages.

**Figure 5:**
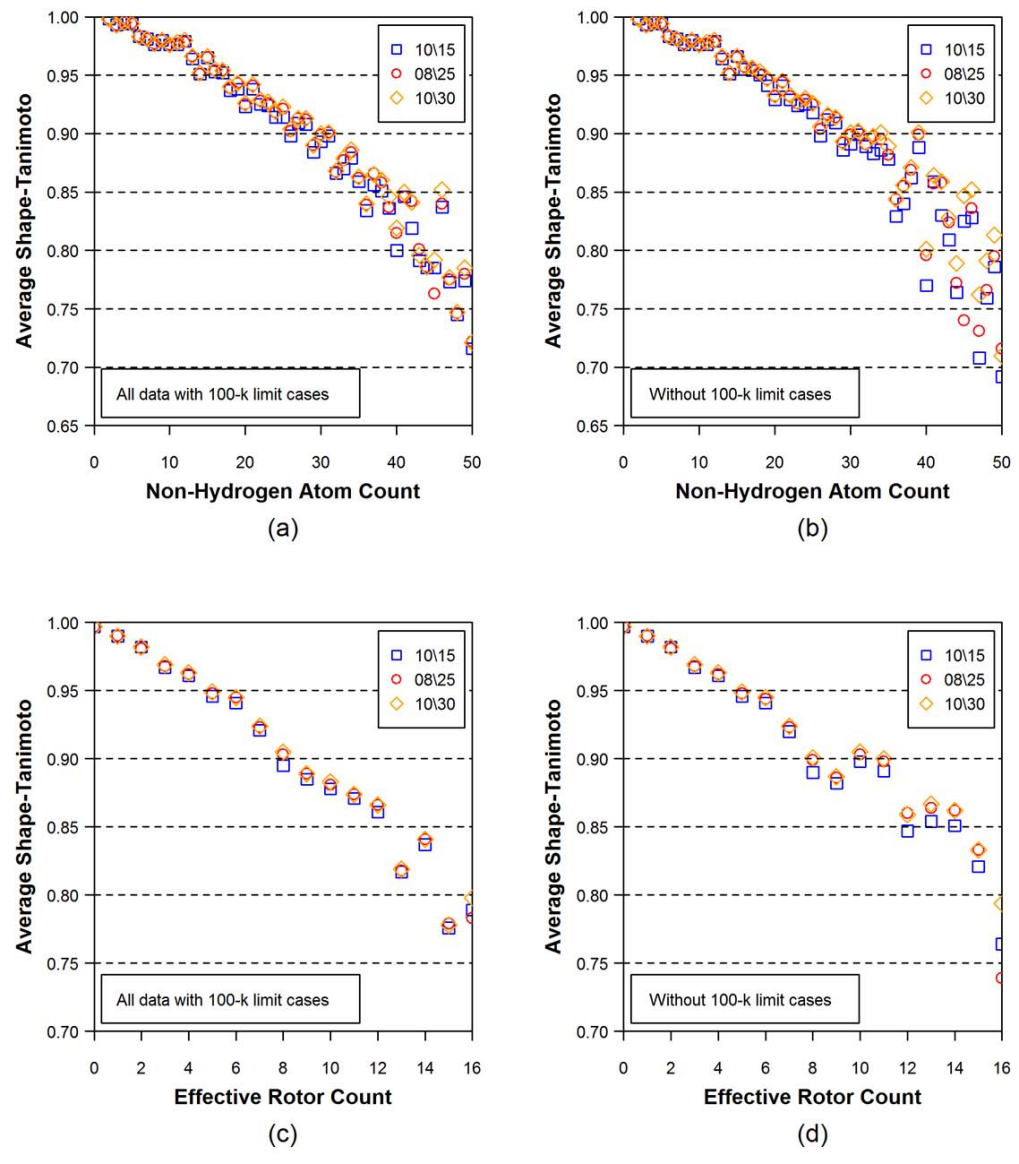
**Average Shape-Tanimoto (ST) values for all 25,972 3D reference structures as a function of non-hydrogen atom count [(a) and (b)] and effective rotor count [(c) and (d)]**. In Panels (b) and (d), the 100-k limit cases, in which the number of conformers reached the maximum number allowed, were removed. The numbers in the legend boxes indicates energy-window values used in the model building (first) and torsion search (second) stages.

Figures 4 and 5 show the average RMSD and the average ST values of the conformer models, respectively, as a function of the non-hydrogen atom count and the effective rotor count for different energy window values. An increase in the non-hydrogen atom count and the effective rotor count causes a linear increase in the RMSD and a linear decrease in the ST values, indicating that the accuracy of conformer model decreases as a function of both the molecular size and flexibility.

**Figure 6:**
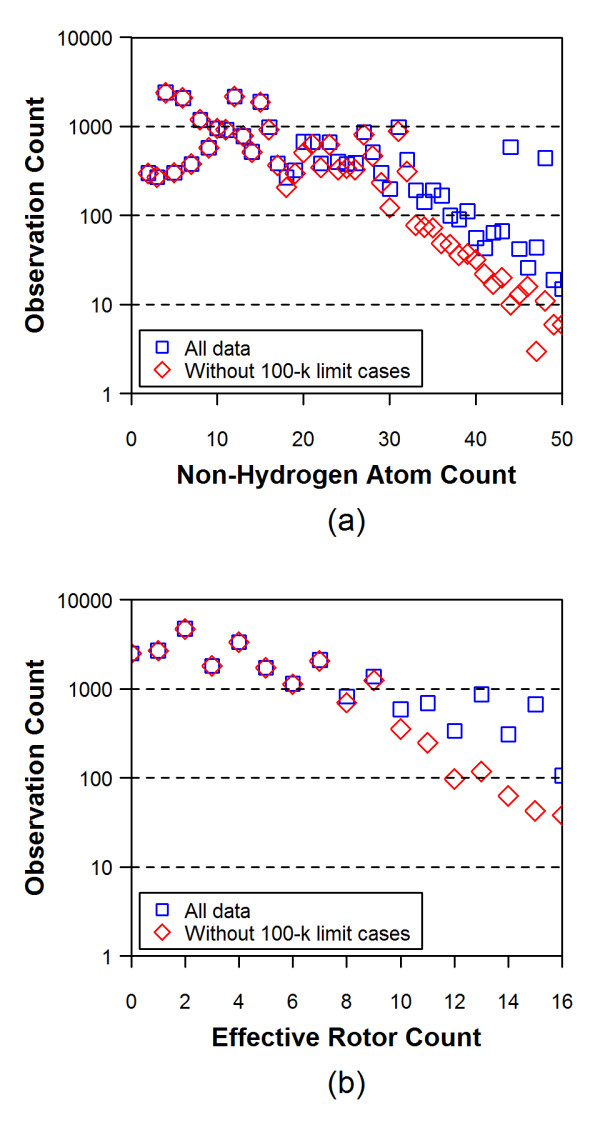
**The effect of the 100-k limit cases upon the distributions of (1) the non-hydrogen atom count and (2) the effective rotor counts**. The MMFF94s_NoEstat force field and the energy window of 5 kcal/mol were used for both the model building and torsion search stages.

**Figure 7:**
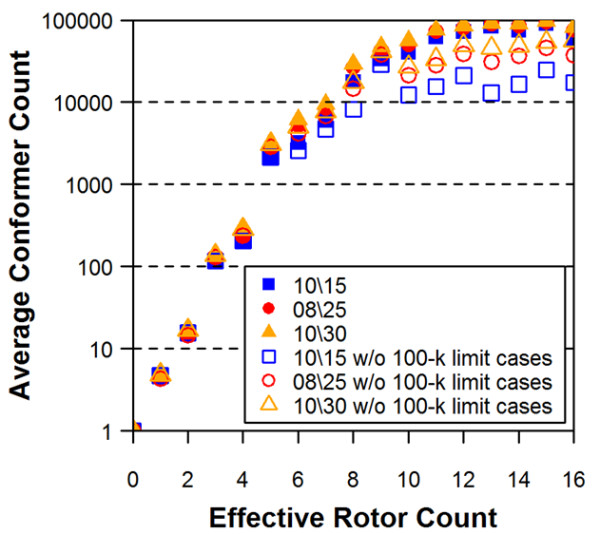
**The average number of conformers for a molecule as a function of the effective rotor count**. The first two numbers in the legend box correspond to the energy-window sizes used in the model building (first) and torsion search (second). While the solid data symbols represent all 25,972 molecules, the open data symbols denote cases in which the 100-k limit cases were removed.

The conformer models that reach the 100-k limit cases may exclude important conformational diversity due to the truncated description of the molecule. Indeed, as Figures 4 and 5 show better accuracy of reproduction when truncated conformer models are excluded. Figure 6 shows effects of the 100-k limit cases upon the distributions of the non-hydrogen atom count and the effective rotor counts, and Figure 7 displays effects of the 100-k limit cases upon the average number of conformers for a molecule as a function of energy window. As one can see in Figure 6, the 100-k limit cases begin appearing for molecules with moderate size and flexibility (*e.g.*, with ~15 non-hydrogen atoms and ~7 effective rotors). As a molecule becomes bigger and more flexible, the OMEGA conformer generation hits the 100-k limit more frequently. Therefore, removal of these cases from the dataset of 25,972 3-D reference structures leaves only a relatively small number of "non-100-k cases" for >30 non-hydrogen atoms and >10 effective rotors, causing a greater variability in the average RMSD and ST in these regions of panels (b) and (d) of Figures 4 and 5. Despite the increased variability, one can still see a slightly noticeable trend of improved accuracy in panel (d) of Figures 4 and 5. One can also see in all panels of Figures 4 and 5 that the energy window for torsion search of 25 kcal/mol gives a clear improvement over 15 kcal/mol for larger values of non-hydrogen atoms and, to a lesser extent, effective rotors. As such, for the remainder of the study 25 kcal/mol will be used for both fragment model and torsion search.

### 4. Prediction of RMSD accuracy of the conformer ensemble

So far, we have found ways to get the best average RMSD and ST accuracy using OMEGA as the conformer generator. Now we are faced with the problem of data reduction, as it is not practical to store or use all conformers produced when considering millions of chemical structures. Naturally, one would like to maximally reduce the conformer count without significantly sacrificing accuracy, e.g., by a minimum RMSD separation between conformers. The lower the separation RMSD used, the greater the count of conformers that must be kept. Conversely, too large of an RMSD separation may reduce the ensemble accuracy. In an ideal world, one would know *a priori *precisely which conformers are needed and discard the rest. In the real world, this is not possible to know; however, if one can reliably predict the RMSD accuracy of a conformer model, then one can devise an RMSD separation value that could be used as a sampling threshold with some statistical assurances that the sampled conformer model should have at least one conformer within the sampling RMSD some significantly large percentage of the time.

**Figure 8:**
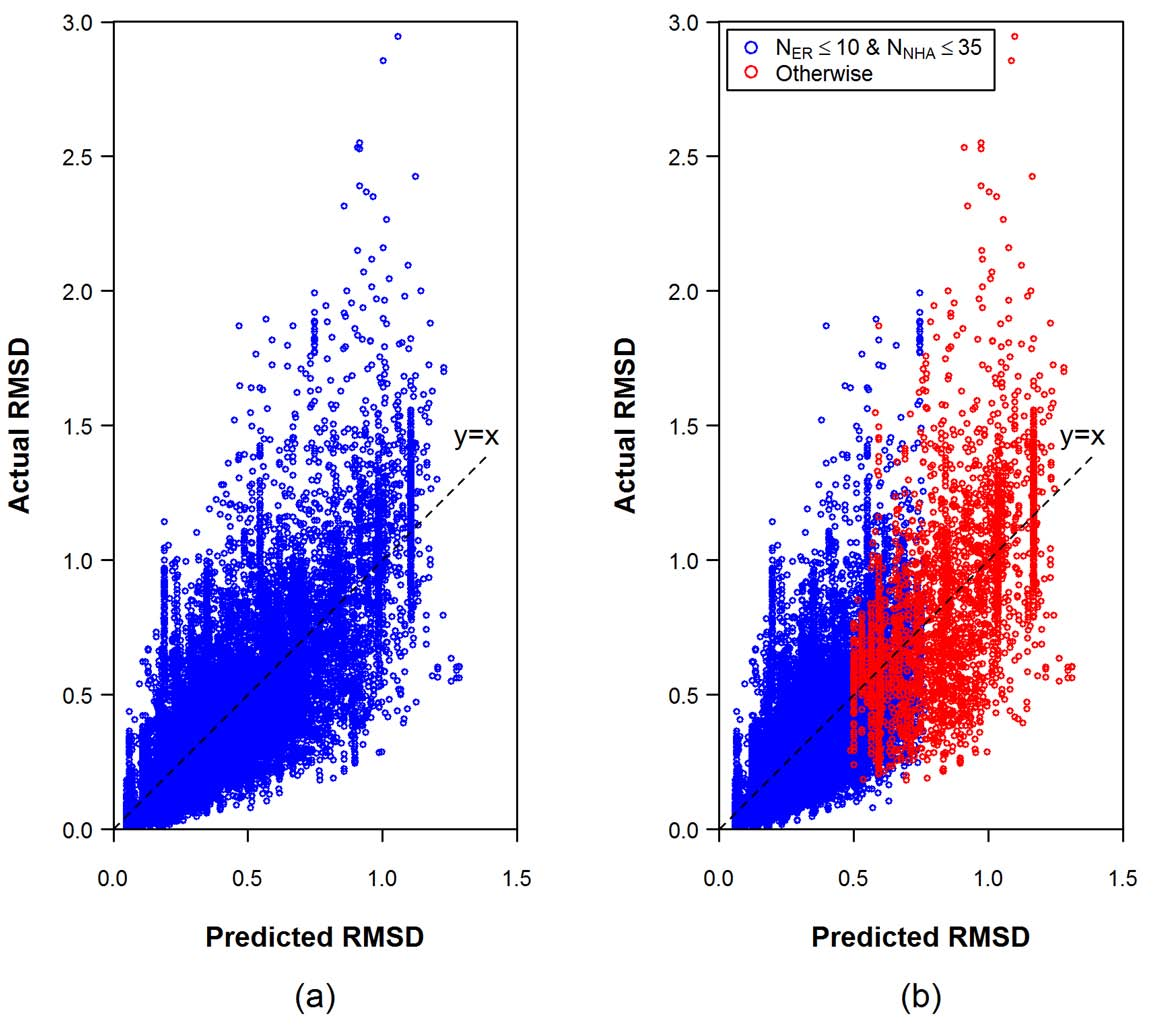
**Comparison of the predicted and actual RMSD of the theoretical conformer models for the 25,972 experimentally determined structures**. While the predicted RMSDs in panel (a) were computed using a single equation [Eq. (4)] for all 25,972 structures, those in panel (b) were computed using two different equations: Eq. (6) for the structures with N_ER_≤10 and N_NHA_≤35 (in blue), and Eq. (7) for otherwise (in red).

With the aim to find a way to derive an RMSD sampling threshold for a given chemical structure, Figures 4 and 5 show that the average RMSD accuracy of the theoretical conformer ensembles has a very linear correlation with both the non-hydrogen atom count and the effective rotor count. Therefore, a linear regression analysis was performed to derive an equation that predicts the RMSD accuracy of the conformer models using just the *N*_*NHA *_and the *N*_*ER*_, yielding Eq. (4).

(4) $$RMSD_{pred}=0.0\text{29 + }0.00\text{99 *}N_{NHA}+\;0.0\text{4}0\text{ *}N_{ER}$$

where *RMSD*_*pred *_is the predicted RMSD of a theoretical conformer ensemble for a molecule, and *N*_*NHA *_and *N*_*ER *_are its non-hydrogen atom and effective rotor counts, respectively. The R^2 ^value of the regression equation for all 25,972 chemical structures was 0.65 and the standard deviation of *RMSD*_*pred *_was 0.19. The correlation between the *RMSD*_*pred *_and the actual RMSD (*RMSD*_*actual*_) is shown in Figure 8.

By design, Eq. (4) overestimates the RMSD value for half of the experimental structures and underestimates the other half. We consider it acceptable to use an RMSD sampling value where 90% of conformer ensembles have the same or lesser RMSD accuracy value on average. This is simply Eq. (4) to which we add the first standard deviation value of 0.19 to yield Eq. (5).

(5) $$RMSD_{pred}=\;0.\text{19 + }0.0\text{29}+0.00\text{99}*N_{NHA}+0.0\text{4}0*N_{ER}.$$

**Figure 9:**
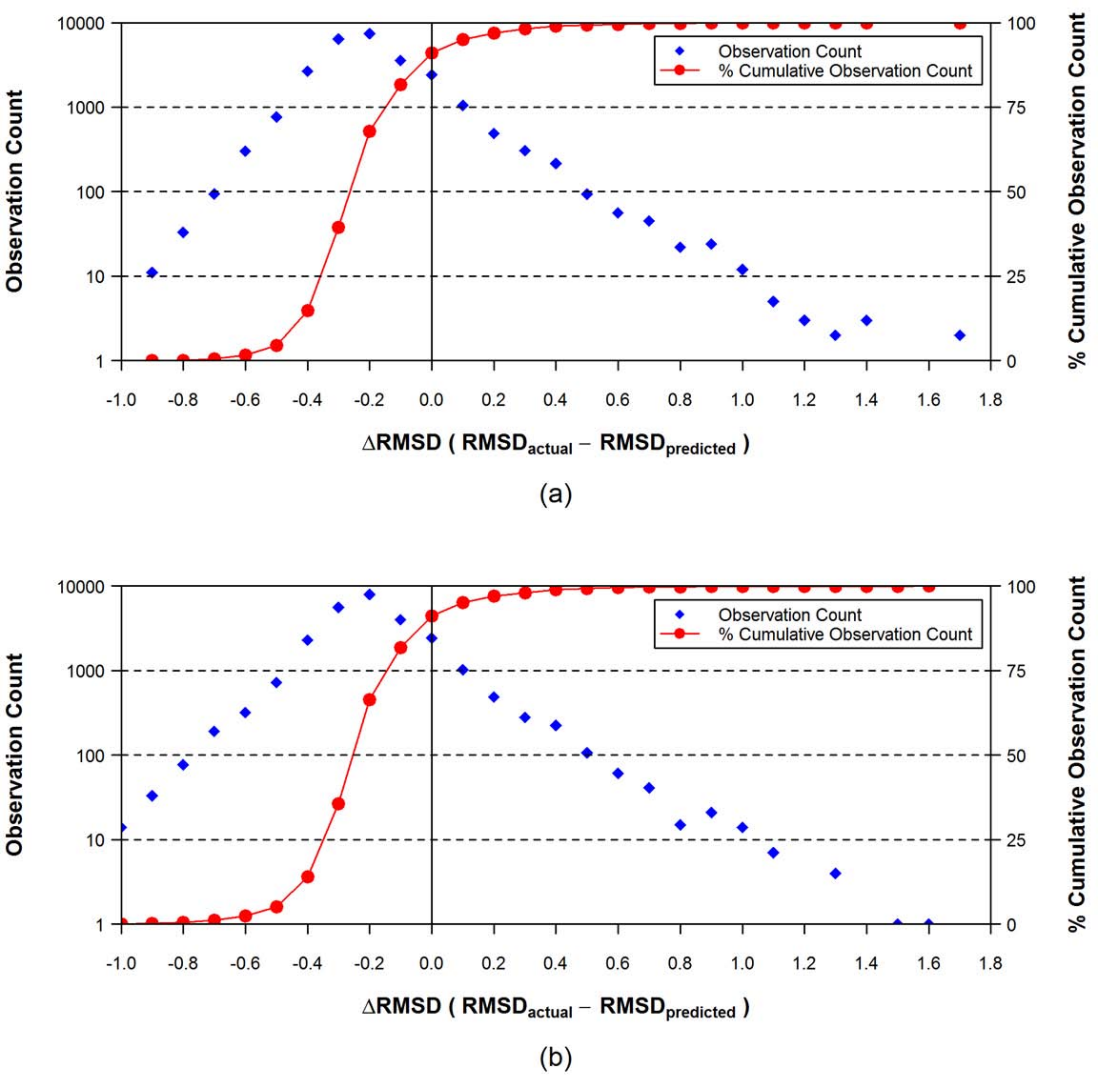
**Frequency distribution of the RMSD differences between the actual RMSD and the predicted RMSD values for the conformer models of the 25,972 experimental structures, binned in 0.1 increments using Eq. (5) for panel (a) and Eqs. (8) and (9) for panel (b). (See text)**.

To highlight this, we plotted in Figure 9 the distribution of the difference between *RMSD*_*actual *_and *RMSD*_*pred *_using Eq. (5), binned in 0.1 increments. Figure 9 shows that Eq. (5) yields an *RMSD*_*pred *_that is greater than or equal to *RMSD*_*actual *_more than 90% of the time.

As shown in Figures 4 and 5, the average RMSD increases and the average ST decreases as a function of both *N*_*ER *_and *N*_*NHA*_, regardless of the inclusion of 100-K limit cases. Given how the variability increases as chemical structures become larger and more flexible, potentially due, in part, to their lower populations, it may be helpful to partition data into separate groups according to their *N*_*ER *_and *N*_*NHA *_values and perform separate regression analyses. A regression analysis of the 22,587 structures with *N*_*ER*_≤10 and *N*_*NHA*_≤35 yields Eq. (6), while a regression analysis of the 3,385 structures with *N*_*ER*_>10 or *N*_*NHA*_>35 yields Eq. (7):

(6) $$RMSD_{pred}=0.0\text{46}+0.00\text{63}*N_{NHA}+0.0\text{5}0*N_{ER}\;\;(\text{for }N_{ER}\leq \text{1}0\text{ and }N_{NHA}\leq \text{35})$$

(7) $$RMSD_{pred}=-0.\text{1}0\text{1}+0.0\text{155}*N_{NHA}\text{+ }0.0\text{35}*N_{ER}\;\;\left(\text{for }N_{ER}\text{>1}0\text{ and }N_{NHA}>\text{35}\right)$$

(8) $$RMSD_{pred}=0.\text{17}+0.0\text{46}+0.00\text{63}*N_{NHA}+0.0\text{5}0*N_{ER}\;(\text{for }N_{ER}\leq \text{1}0\text{ and }N_{NHA}\leq \text{35})$$

(9) $$RMSD_{pred}=0.\text{29}-0.\text{1}0\text{1}+0.0\text{155}*N_{NHA}+0.0\text{35}*N_{ER}\;\left(\text{for }N_{ER}>\text{1}0\text{ and }N_{NHA}>\text{35}\right)$$

The R^2 ^values of the regression formula Eqs. (6) and (7) were 0.52 and 0.33, respectively, and the standard deviation of *RMSD*_*pred *_was 0.17 and 0.29 for Eqs. (6) and (7), respectively. (Attempts to partition *N*_*ER *_and *N*_*NHA *_values using different partitioning schemes had similar R^2 ^results.) In the same manner as used to derive Eq. (5), one can add these first standard deviations to Eqs. (6) and (7) to get RMSD sampling formulas Eqs. (8) and (9), respectively, for the two individual groups. As shown in Figure 8, the *RMSD*_*pred *_values from Eqs. (6) and (7) are comparable with those from Eq. (4), despite the poor R^2 ^values for Eqs. (6) and (7). As shown in Figure 9, where the RMSD sampling values from Eq. (5) are compared with those from Eqs. (8) and (9), the frequency distributions of the difference between *RMSD*_*pred *_and *RMSD*_*actual *_are similar to each other. The *RMSD*_*pred *_value is greater than or equal to *RMSD*_*actual *_for ~91.0% of the time when Eq. (5) is used, and for ~91.6% of the time when Eqs. (8) and (9) are used.

In the recent study of Hawkins *et al. *[11], the quality of theoretical conformations from OMEGA was evaluated by comparing a set of 197 high-quality PDB ligand structures with corresponding OMEGA-generated theoretical conformers. They used the MMFF94_Trunc force field to generate conformers and then reduced their count to a maximum of 200 by sampling. The mean RMSD between the 197 ligand set and their corresponding theoretical conformers was 0.67 Å. According to their bootstrapping tests, the OMEGA-generated conformers are expected 90% of the time to have an RMSD value between 0.647-0.688 Å for chemical structures with similar properties to those tested. The average rotatable bond count and non-hydrogen atom count of this 197 ligand set were 6.3 and 24.4, respectively, indicating that they are, on average, slightly bigger and flexible than the 25,972 set used in our study (4.9 rotatable bonds and 17.1 non-hydrogen atoms on average). With these average counts as the *N*_*NHA *_and *N*_*ER *_values in Eq. (5), RMSD_pred _is predicted to be 0.71 Å, which is comparable to 0.67 Å, the mean RMSD between the 197 ligand set and the corresponding theoretical conformers. Considering that the OMEGA parameters used in both studies were not exactly identical and that we used their reported rotatable bond count rather than effective rotor count, this may suggest that Eq (5) may have applicability beyond the parameter sets used in this study.

## Conclusion

In the present study, theoretical conformer ensembles for 25,972 experimental MMDB ligand molecules were generated using the OMEGA software and the accuracy of the conformer models were analyzed in terms of the non-hydrogen atom pair-wise RMSD and shape similarity ST values between the theoretical conformer models and the corresponding experimental structures.

Effects of different settings for three important parameters (the fragment sampling rate, the type of the molecular force field, and the size of the energy window) that OMEGA uses for conformer generation were investigated to find their optimal settings. The use of a fragment sampling rate greater than the default (= 20.0) did not make a statistically significant change, indicating the default fragment sampling rate is already sufficient. Variation in the fragment force field type was found to provide little benefit. However, the accuracy of the theoretical conformer models was sensitive to the force field type used in the torsion search stage. When used as a torsion search force field, the MMFF94s_NoEstat and MMFF94s_Trunc force fields were found to generate more conformers for a given molecule and also improve the overall accuracy of the theoretical conformer models, compared to the MMFF94s force field but less so as the energy window was increased. In general, using a greater energy window in the model building and torsion search stages resulted in more accurate conformer models. However, a rapid convergence in the overall average RMSD and ST of the conformer models was observed when the energy windows were bigger than 10 kcal/mol for model building and 15 kcal/mol for torsion search. However, for larger and more flexible structures, an energy window of 25 kcal/mol for torsion search gave some noticeable improvement to the overall accuracy for larger and more flexible structures and may be better threshold for general purpose use. The average accuracy as a function of non-hydrogen atom count (size) and effective rotor count (flexibility) was very linear in the ranges considered. A regression equation was developed using these two variables to predict the accuracy of a theoretical ensemble to reproduce the experimental geometry. This equation was subsequently used to provide a RMSD sampling rate to filter conformer models such that 90% of conformer ensembles should have the same or lesser RMSD accuracy value, thus, allowing one to maximize the accuracy of a conformer model while minimizing the count of retained conformers.

## Materials and Methods

### 1. Datasets

The "experimental" 3-D coordinate data set of small molecules used in the present study was downloaded from the Molecular Modeling DataBase (MMDB) [21,22] ligand dataset as available from the PubChem Substance database at NCBI on October 20, 2006. The data set was used to calibrate the parameters used when operating the software generation package OMEGA [19]. Ligands that were too small or too big were discarded by limiting the non-hydrogen atom count to 2 - 50. Ligands too flexible (with a rotatable bond count greater than 15) were also eliminated. This filtering stage resulted in an initial dataset that contained 25,972 non-unique organic 3-D experimental reference structures where a 3-D conformer model could be generated.

### 2. Conformer generation using OMEGA

Essentially, the OMEGA application performs conformer generation in two primary stages: model building and torsion search. In the model building stage, initial molecular structures are constructed by assembling fragment templates, which are generated from fragmentation of the input molecular graph along sigma bonds. In the torsion search stage, OMEGA generates a conformer ensemble using particular rule-based torsion angles that depend on the molecular environment between connecting fragments.

There are a number of adjustable parameters available when performing conformer generation. The effects of three primary parameters upon the accuracy of conformer models generated were evaluated:

(1) -startfact (20 or 50): the argument of this option determines the fragment sampling rate for determination of fragment conformations. Effects of this parameter were studied by trying two values, 20 (default) and 50.

(2) -buildff and -searchff (MMFF94s, MMFF94s_NoEstat, or MMFF94s_Trunc): these specify the type of molecular force field used in the model building and torsion search stages, respectively. The MMFF94s argument tells OMEGA to use the 94s variant of Merck molecular force field. The MMFF94s_NoStat excludes the coulombic terms from the MMFF94s force field and the MMFF94s_Trunc means the MMFF94s force field was truncated by removal of both the coulombic and van der Waals attractive terms.

(3) -ewindow (1-10, 15, 20, 25, or 30): the size of energy window in the model building and torsion search stages in units of kcal/mol. Different values may be specified for the two stages. Various combinations of integer values ranging from one to thirty (default is 10) were used. The energy window indicates the maximum allowed energetic separation from the lowest conformer energy. Higher energy conformers are discarded.

See Table 1 for all non-default parameters used. It is important to note that a maximum of 100,000 (100-k) conformers were generated per chemical structure. This limit is more than adequate for small or inflexible structures but for flexible compounds this limitation is of concern. For example, imagine a chemical structure with nine rotatable bonds where one systematically samples each rotatable bond four times; one would generate 4^9 ^(= 262,144) conformations. Therefore, the effects of these 100-k limit cases upon the overall accuracy of the conformer models is analyzed as a function of the non-hydrogen atom counts and the effective rotor counts. While increasing this 100-k threshold to a larger value was not possible with earlier versions of OMEGA, one can see that increasing the total count of conformers considered by five or ten times would still not be sufficient for many flexible molecules.

To assess the accuracy of reproduction of experimental coordinates as a function of conformer generation parameters modification, two metrics were used: the RMSD of non-hydrogen atoms using the OEChem OERMSD function (with "automorph" detection turned on to allow proper treatment of symmetrically equivalent atoms and "overlay" turned on to allow rotation/translation to yield the lowest possible RMSD value) and the shape-optimized ST using the value reported by ROCS. For each conformer produced for a structure, an RMSD and ST determination were made. The lowest RMSD and greatest ST values per conformer model were used to assess "accuracy" of reproduction for the parameters used.

## Competing interests

The authors declare that they have no competing interests.

## Authors' contributions

EEB performed most of the research and SK wrote the first draft. SHB reviewed the final manuscript. All authors read and approved the final manuscript.
